# Classism and Everyday Racism as Experienced by Racialized Health Care Users: A Concept Mapping Study

**DOI:** 10.1177/00207314211014782

**Published:** 2021-07

**Authors:** Deb F. Mahabir, Patricia O'Campo, Aisha Lofters, Ketan Shankardass, Christina Salmon, Carles Muntaner

**Affiliations:** 1 7938 University of Toronto, Toronto, ON, Canada; 2 MAP Centre for Urban Health Solutions, Toronto, ON, Canada; 3 7985 Women's College Hospital, Toronto, ON, Canada; 4 8431 Wilfrid Laurier University, Waterloo, ON, Canada; 5 518773 Knowledge Translation Li Ka Shing Knowledge Institute, St. Michael's Hospital, Toronto, ON, Canada; 6 Dalla Lana School of Public Health, 7938 University of Toronto, ON, Canada

**Keywords:** classism, concept mapping, everyday racism, institutional racism policy, health care, social class, socioeconomic position

## Abstract

In Toronto, Canada, 51.5 % of the population are members of racialized groups. Systemic labor market racism has resulted in an overrepresentation of racialized groups in low-income and precarious jobs, a racialization of poverty, and poor health. Yet, the health care system is structured around a model of service delivery and policies that fail to consider unequal power social relations or racism. This study examines how racialized health care users experience classism and everyday racism in the health care setting and whether these experiences differ within stratifications such as social class, gender, and immigration status. A concept mapping design was used to identify mechanisms of classism and everyday racism. For the rating activity, 41 participants identified as racialized health care users. The data analysis was completed using concept systems software. Racialized health care users reported “race”/ethnic-based discrimination as moderate to high and socioeconomic position-/social class-based discrimination as moderate in importance for the challenges experienced when receiving health care; differences within stratifications were also identified. To improve access to services and quality of care, antiracist policies that focus on unequal power social relations and a broader systems thinking are needed to address institutional racism within the health care system.

## Introduction

Research has consistently demonstrated the harmful impact of racism on physical and mental health.^1–5^ In Canada, racism remains entrenched within society,^6^ with evidence of health inequities among indigenous, racialized, and immigrant groups.^7,8^ According to the 2016 census data, 51.5 % of Toronto’s population (Canada's most populous city, located in the province of Ontario) are members of racialized groups;^9^ projected estimates suggest a continued increase to 63 % by the year 2031.^10^

Structural racism, an integrated system of policies and laws at the structural or macro level, resulting in racial/ethnic stratification,^11^ is interconnected to the institutional level (eg, hospitals) and to the interpersonal level, resulting in everyday racism.^12^ “Race”/ethnicity, a social construct, is defined in this study as a power-based social relation: a set of social relations that are a subset of the structure of a social system.^13^ Power differences in social relations result in a racial hierarchy and are, in part, the result of oppressive policies and laws.^11–13^ Racialization is understood as the social construction of racial categories that are different and unequal (on the superficial basis of attributes such as skin color) such that they have social, economic, and political consequences.^14^

In Canada, 1 way that systemic racism plays out is through unequal access to or social exclusion from the labor market, resulting in racial stratifications and a racialization of poverty. Within the labor market, irrespective of educational attainment, there is a history of an overrepresentation of racialized groups, including racialized immigrants, in low-income jobs, forms of precarious work, and unemployment.^14–17^ Moreover, more than half (51.6 %) of recent immigrants are overqualified for their jobs based on their level of education.^18^ Racialized women are 48 % more likely to be unemployed and, when employed, earn an income of 55.6 % as compared to nonracialized men.^19^ This social exclusion from the labor market in Canada is linked to poor health.^20^

More recently, the COVID-19 pandemic has further exacerbated the socioeconomic hardships faced by racialized groups. Recent research indicates that in Canada, the pandemic generally has had a stronger impact on the ability of racialized groups to meet financial obligations or essential needs.^21^ This suggests that racialized groups may need targeted interventions to address their health care needs.

Social class, defined as a power-based social relation, refers to employment relations in the labor market focusing on the social division that arises due to the ownership and nonownership of productive assets.^22^ Over the past 2 decades, few studies have examined occupational social class (hereafter, social class).^23^ Of these studies, findings have demonstrated that social class and “race”/ethnicity are linked to health inequities.^24–26^ Evidence has also demonstrated that the impact of racism on health can occur *independent* of social class for people in both working-class (nonprofessional) and nonworking-class (professional) occupations.^27^

As compared to the social class concept, most studies examining health inequities use socioeconomic position (SEP). SEP is a gradational concept and refers to the technical aspects of work whereby occupations are ranked hierarchically.^22^ Studies examining health inequities for racialized groups have established a link between poor health and a low SEP;^28–31^ however, studies have also demonstrated that the impact of racism on health is also independent of SEP.^32,33^

In Canada, the health care system is structured around a biomedical model or a biological essentialism of service delivery and a policy of cultural competence; this has implications for racialized groups receiving health care. In viewing “race” as a biological construct, the belief that racial/ethnic groups are less sensitive to pain as compared to nonracialized groups is still held by some health care providers (HCPs).^34^ This belief in biological differences has had an impact on racialized patients. Although in Canada, the data on racialized health care experiences are limited,^35,36^ systematic reviews have demonstrated that in North America, racial/ethnic groups are undertreated for acute, chronic, and palliative pain; this undertreatment occurs across the lifespan and various treatment settings.^37–39^ Another systematic review found that African-Americans are less likely to be prescribed and to receive pain medication (nonopioid and opioid) in health care settings.^40^

Cultural competence in the health care setting focuses on an HCP's individual behavior when providing patient care. Critical analyses of cultural competence critique this policy as a form of racism for its focus on essentializing culture and for failing to theorize power and systems of oppression.^41–44^ Despite these critiques and evidence demonstrating its limited effectiveness in patient health outcomes and health care access and utilization,^45^ the policy of cultural competence continues to guide practice in the health care setting.

According to a recent systematic review, absent within the extant literature is the contribution of historic or current federal and institutional regulations, policies, and practices toward maintaining institutional racism.^46^ To support identifying mechanisms connected to policy processes, a political economy model of society—which considers the distribution of power and how economic, political, and cultural (or ideological) structures or context influence policy^47–49^—is used in this study. Within this view, individual stratification in society is the result of unobserved, macro-level mechanisms based on oppressive histories and underlying asymmetrical or unequal power social relations (racism, classism, and sexism).^50^ Social mechanisms (at the micro level) are the underlying process of reasoning, beliefs, preferences, and collective norms that lead to decisions, choices, and outcomes; these mechanisms are shaped and constrained by social context,^51^ including unequal power social relations.^13^

The purpose of this study is to understand the effect of health care policies and practices for different racial/ethnic groups at the level of the individual by identifying mechanisms of exclusion and, in particular, classism and everyday racism. Specifically, this study examines how racialized health care users (HCUs) feel or experience exclusion in the health care setting of Toronto and the Greater Toronto Area (GTA); and whether these experiences of exclusion differ within stratifications such as social class, gender, and immigration status (immigrant vs Canadian born).

## Methods

For this study, a concept mapping (CM) study design^52^ with a participatory approach^53^ was selected to support an understanding of classism and everyday racism in the health care setting and, by extension, institutional racism. CM was first used in public health by O’Campo and colleagues,^54^ who also adapted the CM design to support a participatory approach. CM supports the inclusion of a range of participant-generated ideas and, with the application of multidimensional scaling and hierarchical cluster analysis, provides an objective, replicable structure to the qualitative data. CM supports an analysis of how identified themes relate to one another; the simultaneous exploration of multiple themes, including the differences within or between groups; and the possible relationships between themes.^53^ CM is a semiqualitative design consisting of 4 data collection activities: brainstorming, sorting, rating, and mapping. Although detailed descriptions of CM are described elsewhere,^52,53^ we briefly describe CM steps/processes specific to our study below.

## Participants

As recommended by Kane and Trochim,^52^ this study used purposive sampling for heterogeneity to support the selection of participants with known salience specific to the subject matter and was not intended to ensure generalizability. Participants were comprised of HCUs and HCPs. For HCUs, participant eligibility was based on the following criteria: a negative experience in Toronto or the GTA health care setting within the past 5 years, age 16 years or older, and able to write in English at a self-identified “very good,” “good,” or “intermediate” level. For HCPs, eligibility was based on at least 1 year of practice experience working in Toronto or the GTA as a frontline provider (eg, nurse, doctor, social worker, pharmacist).

Prior to data collection, all research protocols, participant information sheets, and appropriate reimbursements were submitted and approved by the University of Toronto's Research Ethics Board. For each activity, participant informed consent was obtained. The brainstorming, sorting, and rating activities were completed online. For the mapping activity, data collection took place in person at the MAP Centre for Urban Health Solutions, located in central downtown Toronto.

## Data Collection Activities

### Brainstorming

During the brainstorming activity, participants generated qualitative data or statements using a focal prompt; statements are based on the participant's experiences. To identify mechanisms of how, in general, experiences of exclusion occur, the focal question used in this study was: “One way in which patients feel disrespected or feel mistreated when seeking good quality health care (service) is…?” For HCUs, the intent was to generate statements of mechanisms in which they had experienced disrespect or mistreatment when receiving health care. For HCPs, the intent was to generate statements of mechanisms based on their knowledge of processes that may result in disrespect or mistreatment for HCUs when receiving health care.

### Sorting

During the sorting activity, participants individually sorted statements into conceptually similar piles. Multidimensional scaling was used to represent the participants' aggregated sort data onto a 2-dimensional configuration or map of statements. Hierarchical cluster analysis was used to group the participants’ statements into distinct conceptually similar clusters of statements in order to create the cluster map.^52^ These statistical analyses were performed using the concept systems software.

### Rating

For the rating activity, based on the rating question selected and using a scale from 1 to 5, participants rated each statement on the degree of importance based on their perceptions or opinions. We used the racialized HCU data for the following rating questions: “Rate how important discrimination based on ‘race’/ethnicity is as a reason for experiencing these challenges” and “Rate how important socioeconomic position (social class) is as a reason for experiencing these challenges.” Responses were used to make distinctions about the degree of importance for each statement based on the racialized HCUs' perceptions or opinions of racial/ethnic and SEP/social class-based discrimination in Toronto's health care setting. In order to compare qualitative differences, the ratings for individual statements and aggregated cluster averages were divided into 3 categories of importance: “high” (rated 3.8 or higher), “moderate” (rated 3.7–2.9), and “low” (rated 2.8 or lower).

### Mapping

Using the cluster map, during the mapping activity, participants reviewed statements in each cluster to confirm that the statements located within the cluster were conceptually similar. After a participant group discussion, participants identified and agreed on a theme for each cluster of statements. Next, after reviewing the participant thematically labeled clusters, as the researchers of this study, we identified and thematically labeled the conceptual regions on the cluster map.

### Identifying Within Stratification Differences

To examine differences within stratifications, we used pattern match graphs. Based on the rating activity data, a correlation coefficient *r* value of 1.0 indicates complete agreement in racialized HCUs’ perceptions and opinions (depicted as horizontal lines between clusters), whereas an *r* value of −1.0 indicates complete disagreement (depicted as diagonal lines). For the stratifications, we compared racialized working-class HCUs to racialized nonworking-class HCUs, racialized female HCUs to racialized male HCUs, and racialized immigrant HCUs to racialized Canadian-born HCUs.

### Identifying Importance for Action/Change

To identify statements rated high in importance for action/change, we used the go-zone data from racialized HCUs. The go-zone is a simple bivariate plot divided into 4 quadrants and, in this study, identifies the ratings of statements based on 2 rating questions: “Rate how important discrimination based on ‘race’/ethnicity is as a reason for experiencing these challenges” and “Rate how important each statement is for action or change.” Each statement within a cluster falls into 1 of 4 quadrants. The upper right quadrant, known as the “go-zone,” contains the statements that were highly rated on the rating questions selected.

### Analytic Categories

Participants were asked to self-identify based on 4 stratifications: “race”/ethnicity, social class, gender, and immigration status. For the “race”/ethnicity stratification, participants were asked to self-identify using the categories developed by the Canadian Employment Equity Act. Because these categories are used by Statistics Canada, in this study, these categories are used in order to situate findings within the Canadian empirical literature.

For the social class stratification, we used Wright's^55^ contemporary conceptualization of social class; categories used were “working-class” and “nonworking-class” HCUs. The working-class group consisted of participants who self-identified as “working,” whereas the nonworking-class group consisted of participants who self-identified as “supervisor/manager” or “owner of business.”

## Results

### Sample Composition

For all CM activities, racialized and nonracialized HCUs and HCPs participated. For the rating activity, racialized HCUs self-identified in the following racial/ethnic categories (in alphabetical order): Black, Chinese, Korean, Latin American, South Asian, Southeast Asian, West Asian, White, and other. Of the 72 participants who completed the rating activity, 41 participants identified as racialized HCUs. Of the racialized HCUs, for the gender and immigration stratification, 25 participants identified as female and 22 identified as Canadian born; for the social class stratification, 16 identified in the working-class occupation, 10 identified in the nonworking-class occupation, and 15 identified as other (eg, student, unemployed, retired).

### Identification of Thematic Clusters

**Figure 1.**
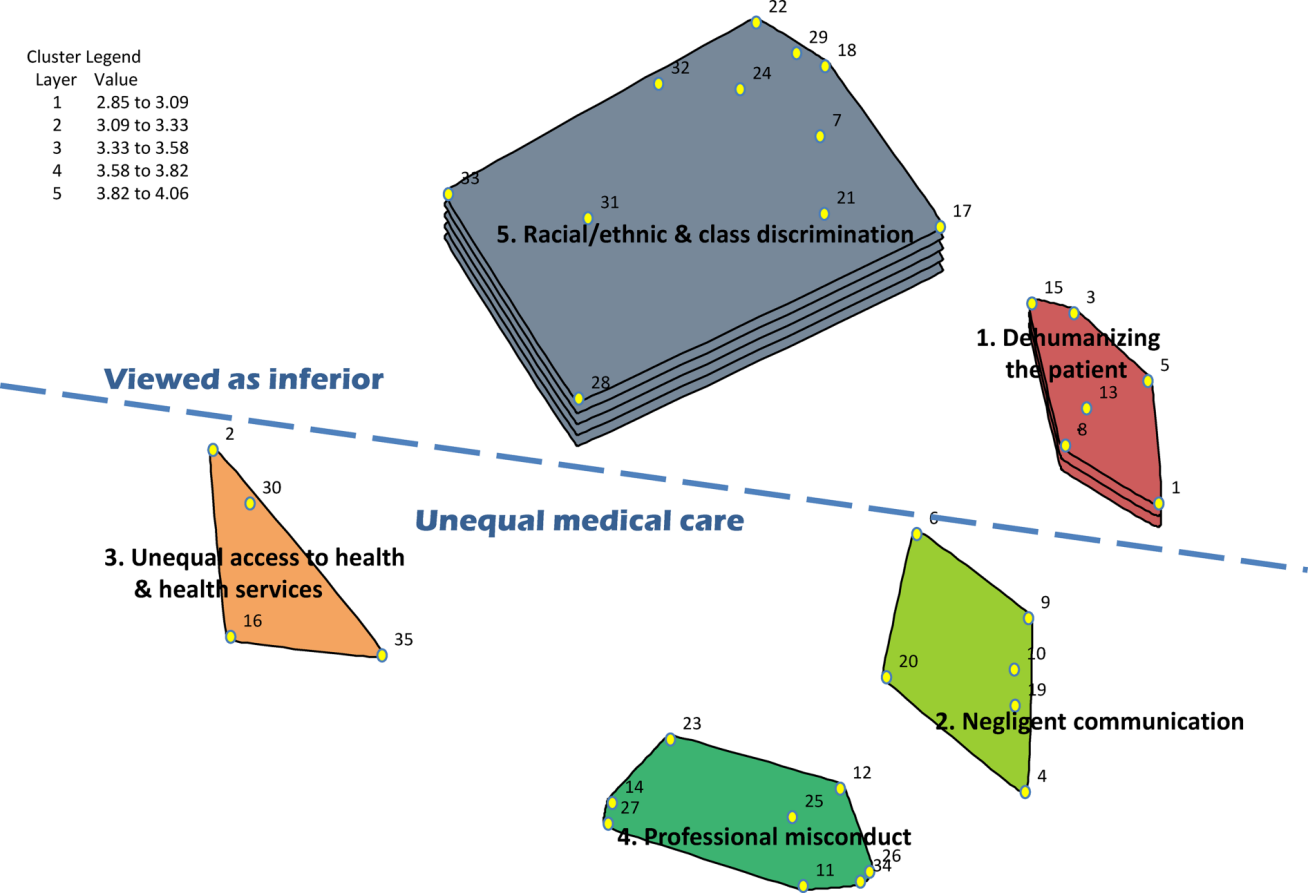
Cluster map with ratings for “race”/ethnic-based discrimination as reported by racialized health care users.

From the brainstorming activity, participants generated 35 unique statements or mechanisms of disrespect or mistreatment. During the sorting activity, participants sorted these statements into conceptually similar piles. Next, participants identified and thematically labeled 5 unique clusters of statements during the mapping activity. From these clusters, 2 conceptually distinct regions were identified and labeled with higher level themes: “Viewed as inferior” and “Unequal medical care” (Figure 1). In CM, a good stress value is <0.36.^56^ The stress value for this study was 0.18, meaning that participants generally agreed upon the location of statements within clusters and the location of clusters on the cluster map.

The “Viewed as inferior” conceptual region, located in the top section of the cluster map, is dominated by statements of experiences that describe activities or behaviors generally pertaining to interpersonal interactions by health care personnel, in which the patient, the patient’s family, or their needs are viewed as inferior. This region consists of 2 clusters: “Racial/ethnic and class discrimination” and “Dehumanizing the patient.”

The “Unequal medical care” conceptual region, located in the lower section of the cluster map, is dominated by statements of experiences that describe structural conditions, or activities and behaviors generally pertaining to interpersonal interactions that involve unequal medical access and treatment. This area consists of 3 clusters: “Unequal access to health and health services,” “Negligent communication,” and “Professional misconduct.”

### Ratings for Thematic Clusters

For racialized HCUs, the aggregated cluster average rating for “race”/ethnic-based discrimination was moderate to high (2.9–4.1) (Figure 1). For SEP/social class-based discrimination, the average rating was moderate (2.9–3.7) (Table 1). For both “race”/ethnic- and SEP/social class-based discrimination, the “Racial/ethnic and class discrimination” cluster had the highest cluster rating (4.1 and 3.7, respectively); this cluster also had the highest rated statements for both forms of discrimination. For “race”/ethnic-based discrimination, the highest rated statements, in order, were “when the White male health care provider continuously picks on the non-White patient” and “when the White health care provider talks to the patient as if they are uneducated.” For SEP/social class-based discrimination, the highest rated statement was “when health care providers or health care staff look down on the patient because of their appearance”; 2 statements had the second-highest rating: “when the patient is looked down on by the health care provider or health care staff for using public transportation” and “when a patient on social assistance is treated in a separate area with fewer resources.”

**Table 1.** Results for “Race”/Ethnic and Socioeconomic Position-based Discrimination as Rated by Racialized Health Care Users.

Cluster	Statement	“Race”/Ethnicity Rating	Socioeconomic Position Rating
Cluster 1: Dehumanizing the patient		Moderate	Moderate
	When the health care provider is disrespectful^15^	High	Moderate
	When the health care provider belittles or talks down to the patient^3^	High	High
	When the health care provider does not show empathy or sympathy^13^	Moderate	Moderate
	When the health care provider will not listen to the patient or pretends that they do not hear the patient^1^	Moderate	Moderate
	When health care provider is impatient with the family after the patient dies^5^	Moderate	Moderate
	When the health care provider or health care support staff is impatient with the patient^8^	Moderate	Moderate
Cluster 2: Negligent communication		Moderate	Moderate
	When the patient's symptoms are ignored or not taken seriously^10^	Moderate	Moderate
	When the health care provider does not consider the patient's concerns about the plan of treatment^20^	Moderate	Moderate
	When the health care provider willfully misunderstands the patient's concerns^9^	Moderate	Moderate
	When the health care provider lies to the patient^19^	Moderate	Moderate
	When the health care provider does not listen to patient's medical history before prescribing medication^4^	Moderate	Moderate
	When the health care support staff places the patient's phone call on hold and then disconnects them^6^	Low	Low
Cluster 3: Unequal access to health and health services		Moderate	Moderate
	When there is little or no access to language interpreters^30^	High	Moderate
	When a patient cannot get access to government-funded assist programs because of where the patient lives^2^	Moderate	High
	When the health care provider tells the patient that they cannot keep them as their patient because they have enough patients^35^	Low	Moderate
	When the patient cannot make an appointment to see their health care provider within a two-week time frame^16^	Low	Low
Cluster 4: Professional misconduct		Moderate	Moderate
	When the patient's pain is not treated^12^	Moderate	Low
	When the health care provider does not provide a referral to see a health care specialist^14^	Moderate	Moderate
	When the patient is discharged prematurely from the hospital^27^	Moderate	Moderate
	When the health care provider does not complete a proper assessment^34^	Moderate	Moderate
	When a patient's message for the health care provider is not relayed by the health care support staff^23^	Low	Low
	When the health care provider does not provide the requested information^25^	Low	Low
	When the health care provider does not read the patient's medical history, resulting in negligent care^26^	Low	Moderate
	When the health care provider does not provide the correct treatment^11^	Low	Low
Cluster 5: Racial/ethnic and class discrimination		High	Moderate
	When the White male health care provider continuously picks on the nonWhite patient^22^	High	Moderate
	When the White health care provider talks to the patient as if they are uneducated^29^	High	High
	When the health care provider wrongly assumes that the patient does not speak English^32^	High	Moderate
	When the patient feels disrespected and not listened to by health care providers because of language issues^31^	High	Moderate
	When health care providers or health care staff look down on the patient because of their appearance^18^	High	High
	When health care provider is unfamiliar with different religious or cultural practices in caring for a loved one who has died^28^	High	Moderate
	When the patient’s concern is thought of by the health care provider as being superstitious^21^	High	Moderate
	When the health care provider engages in victim blaming^17^	High	High
	When the patient is looked down on by the health care provider or health care staff for using public transportation^24^	Moderate	High
	When a patient on social assistance is treated in a separate area with fewer resources^33^	Moderate	High
	When the patient is wrongly judged to be “drug seeking”^7^	Moderate	High

Note. The rating levels were divided into categories: “high” (statements rated 3.8 or higher), “moderate” (statements rated 3.7–2.9), and “low” (statements rated 2.8 or lower).

The second highest rated cluster for “race”/ethnic- and SEP/social class-based discrimination was “Dehumanizing the patient.” For “race”/ethnic-based discrimination, the “Dehumanizing the patient” cluster average was 3.6; within this cluster, for “race”/ethnic-based discrimination, the highest rated statement was “when the health care provider is disrespectful,” and the second highest was “when the health care provider belittles or talks down to the patient.” For SEP/social class-based discrimination, the rating for the “Dehumanizing the patient” cluster was 3.5; within this cluster, the highest rated statement for the SEP/social class-based discrimination was “when the health care provider belittles or talks down to the patient”; the second highest was “when the health care provider is disrespectful.”

### Within Stratification Differences

**Figure 2.**
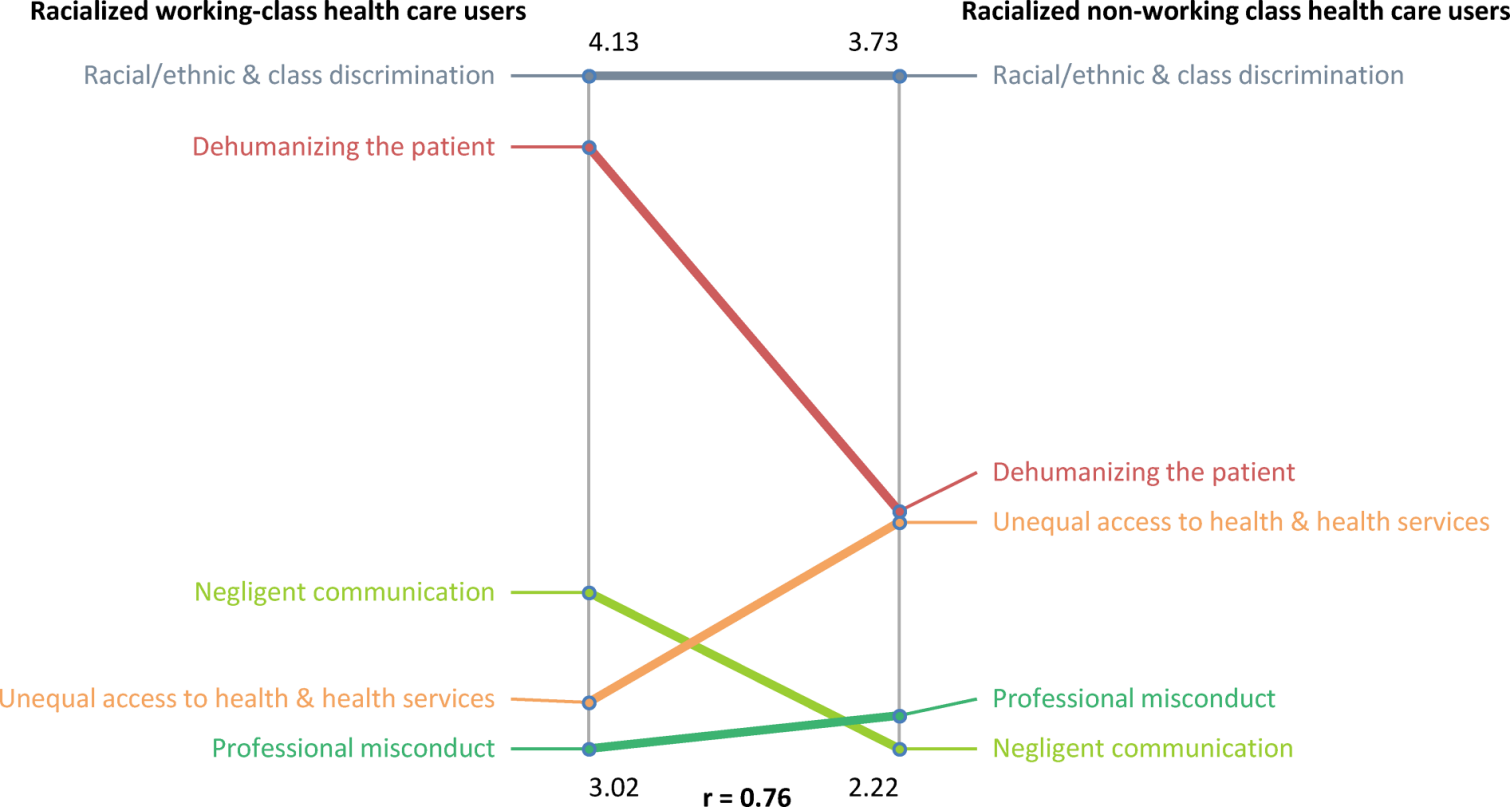
Pattern match comparison between racialized working-class health care users and racialized nonworking class health care users on “race”/ethnic-based discrimination in Toronto's health care system.

To examine whether these experiences of exclusion differ within stratifications such as social class, gender, and immigration status, we used a pattern match graph for each stratification. For the social class pattern match (Figure 2), the correlation coefficient at the cluster level was *r* = .76; this means there is a strong relationship between what racialized working-class HCUs believe and what racialized nonworking-class HCUs believe to be the most important clusters to demonstrate “race”/ethnic-based discrimination as a reason for the challenges experienced when receiving health care. Both groups ranked “Racial/ethnic and class discrimination” as the most important cluster; however, racialized working-class HCUs had a higher cluster average (4.13), as compared to racialized nonworking-class HCUs (3.73). Both groups also ranked “Dehumanizing the patient” as the second most important cluster; racialized working-class HCUs had a higher rating for this cluster. Additionally, racialized working-class HCUs had a higher rating or cluster average for the “Negligent communication” and “Unequal access to health and health services” clusters.

**Figure 3.**
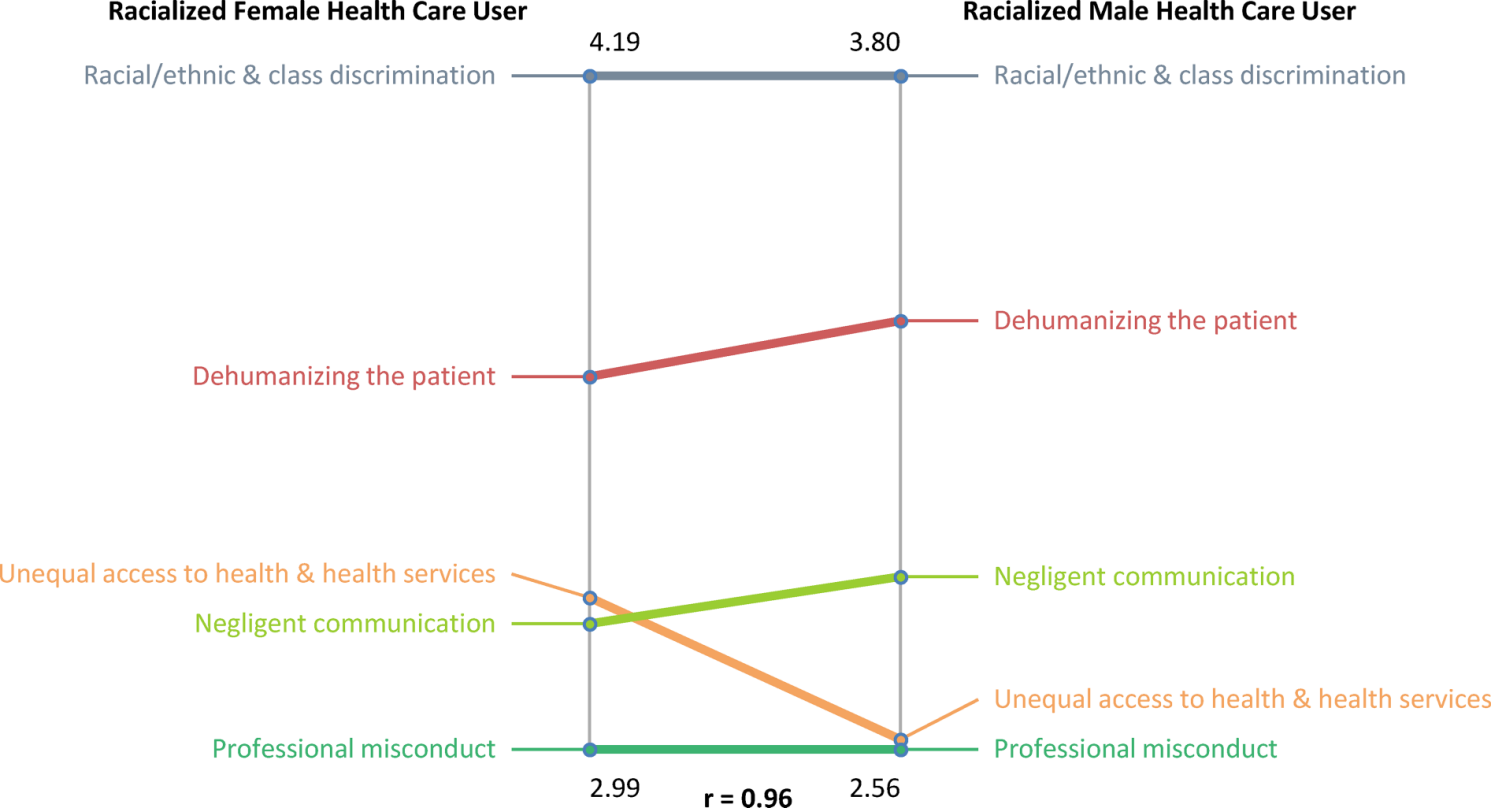
Pattern match comparison between racialized female health care users and racialized male health care users on “race”/ethnic-based discrimination in Toronto's health care system.

For the gender pattern match (Figure 3), the correlation coefficient was *r* = .96; that means there is a strong relationship in beliefs or opinions between racialized female and male HCUs. Both groups agreed on the 2 most important clusters to demonstrate “race”/ethnic-based discrimination. The “Racial/ethnic and class discrimination” cluster was ranked as the most important, with racialized female HCUs having a higher cluster average (4.19). Both groups ranked “Dehumanizing the patient” as the second most important cluster; racialized male HCUs had a higher cluster average. Overall, racialized female HCUs had a higher cluster average for 3 of 5 clusters: “Racial/ethnic and class discrimination,” “Unequal access to health and health services,” and “Professional misconduct.”

**Figure 4.**
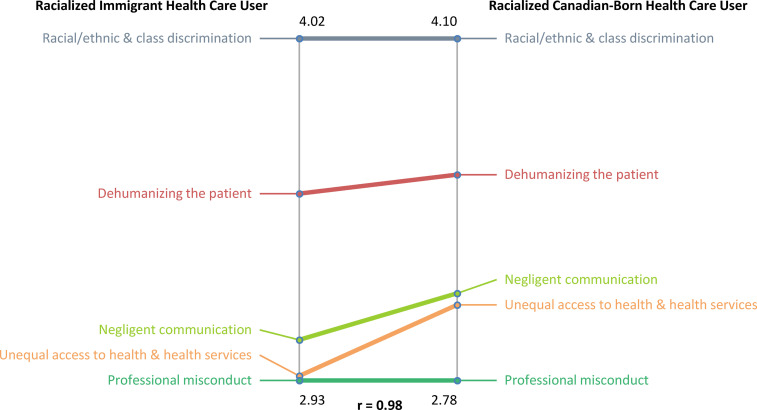
Pattern match comparison between racialized immigrant health care users and Canadian-born health care users on “race”/ethnic-based discrimination in Toronto's health care system.

For the immigration status pattern match (Figure 4), the correlation coefficient was *r* = .98; there is a strong relationship in beliefs or opinions between racialized immigrant and Canadian-born HCUs. Both groups ranked “Racial/ethnic and class discrimination” as the most important cluster, with a higher cluster average (4.10) reported from racialized Canadian-born HCUs. Although all 5 clusters were ranked in the same order of importance, the racialized Canadian-born HCUs had a higher cluster average for the first 4 clusters. Put differently, racialized immigrant HCUs had lower averages for the first 4 clusters.

### Priorities for Action/Change

Overall, racialized HCUs rated clusters in the “Unequal medical care” conceptual region of the cluster map as a priority for action/change. Additionally, statements in the go-zone for the “Unequal access to health and health services” cluster identified 2 additional health care policy processes; these were “when a patient cannot get access to government-funded assist programs because of where the patient lives” and “when there is little or no access to language interpreters.” Both of these statements, as rated by racialized HCUs, had relatively higher ratings for both “race”/ethnic-based discrimination and action/change, as compared to other statements within this cluster.

## Discussion

Systematic reviews continue to demonstrate HCP implicit racial bias.^57,58^ By focusing on how this occurs, studies have recently identified high-level mechanisms of racism in the Canadian health care system for the indigenous population^59^ and in Europe, for racialized groups.^59^ Our CM study adds to the literature by identifying micro-level mechanisms or how experience of classism and everyday racism occurs in Toronto's health care system for racialized HCUs and differences in experiences within social stratifications.

In this study, the labeled cluster map consists of 5 unique clusters of statements or mechanisms and 2 conceptual regions. The clusters from both the “Viewed as inferior” and “Unequal medical care” regions represent the *collective* experiences of participants when receiving health care; simply put, these 2 regions are not mutually exclusive. For example, when receiving health care, a patient may experience “when the health care provider engages in victim blaming” (ie, blaming the individual for poor health) (in the “Viewed as inferior” region) followed by “when the health care provider does not provide a referral to see a health care specialist” (in the “Unequal medical care” region). These mechanisms at the microlevel are the underlying process of reasoning, beliefs, preferences, and collective norms that lead to decisions, choices, and ultimately outcomes (eg, inequities in health care).^51^ Microlevel everyday racism is linked to the activation of underlying power social relations and is interconnected to the mesolevel or institutional level (ie, health care system and labor market) and to the macrolevel or sociopolitical structure.^12^

In this section, we will first discuss experiences of classism and everyday racism as reported by racialized HCUs, followed by how these experiences differ based on social class, gender, and immigration status. Findings are situated within the current literature; in keeping with a political economy model of society, findings are also linked to the local socioeconomic and political context.

### Racialized HCU Experiences

For racialized HCUs, the aggregated cluster averages for “race”/ethnic-based discrimination ranged from moderate to high, meaning that “race”/ethnic based discrimination was central to the challenges experienced in the health care setting. In other words, racialized HCUs reported “race”/ethnic-based discrimination as largely contributory to the challenges experienced when receiving health care. Additionally, statements from the “Viewed as inferior” and “Unequal medical access” region both contained statements that were rated moderate to high, indicating that for racialized HCUs, “race”/ethnic-based discrimination impacts both quality of care and access. These findings align with a landmark US report by the Institute of Medicine,^38^ which identified that implicit racial bias and stereotyping of racial/ethnic groups impact the treatment of patients in 3 central areas of access and quality: unequal treatment/access, lower quality of health care, and undertreatment of pain. Findings also align with a literature review that identified emerging evidence of unequal treatment/access and lower quality of health care for racialized groups in Canada.^35^

For SEP/social class-based discrimination, the aggregated cluster average ranged from moderate to high, with no clusters rated as low. The similarity in cluster averages, when compared to “race”/ethnic-based discrimination cluster averages, suggests that for racialized HCUs, “race”/ethnic- and SEP/social class-based discrimination are interconnected. This aligns with previous research findings that “race”/ethnicity is generally linked to a lower SEP (and poor health outcomes).^30,31^

In this study, racialized HCUs rated the statement “when the patient's pain is not treated” as moderate for “race”/ethnic-based discrimination. This finding is consistent with a Toronto Public Health study^61^ that identified that racialized groups were more likely to have pain or discomfort. More generally, reviews of the literature have demonstrated that racialized groups are undertreated for pain across the lifespan^37,39^ and less likely to be prescribed and to receive pain medication (nonopioid and opioid) in health care settings.^40^

Racialized HCUs rated “when the patient is discharged prematurely from the hospital” as moderate for “race”/ethnic-based discrimination. When discharged prematurely from the hospital, racialized HCUs may not have their continued health care needs met. Premature hospital discharges occur in an economic context in which racialized groups within Canadian society are relegated to lower paying jobs and, therefore, income,^16,17,62^ while paying more for health care coverage and services. Research specific to Ontario reveals a decrease in health care coverage (e.g., home care services) and an increase in user charges for some health services (e.g., physiotherapy).^63^ Furthermore, a system whereby publicly funded home care services are now managed by a few large, for-profit agencies has resulted in diminished services and access for immigrant, racialized, and non-English-speaking groups.^64^

### Differences in Experiences Within Stratifications

From the pattern match graphs, findings demonstrate a strong relationship in terms of clusters that are believed to be most important in demonstrating “racial”/ethnic-based discrimination. Participants generally ranked the clusters in the same order of importance; however, in terms of cluster ratings or aggregated cluster averages, differences were identified within stratifications.

For the social class stratification, racialized nonworking-class HCUs had a higher rating for the first 3 ranked clusters. The clusters “Racial/ethnic and class discrimination” and “Dehumanizing the patient” are located in the “Viewed as inferior” conceptual region of the cluster map, whereas the cluster “Negligent communication” is in the “Unequal medical access” region. This suggests that for racialized working-class HCUs, their “race”/ethnicity and social class are linked to their experiences of a lower quality of care and unequal medical access.

Experiences of SEP/social class-based discrimination as reported by racialized HCUs are occurring in a socioeconomic and political context of increasing poverty among racialized groups. Recent research demonstrates that in Canada, racialized groups continue to be excluded from the labor market.^65,66^ In Toronto, members of racialized groups represent 62 % of all persons living in poverty.^62^ Furthermore, almost two-thirds of the “working poor” are racialized workers.^67^

Findings from our study also indicate that SEP/social class intersects with “race”/ethnic-based discrimination in the health care setting, which may further impact the health of racialized working-class HCUs. With evidence of health inequities at the intersections of “race”/ethnicity and social class,^24–26^ researchers continue to correctly argue for the use of a theoretical framework in research that recognizes that social class interacts with “race”/ethnic-based discrimination to determine racial inequities in health.^23^ In order to improve the health care system, using a theoretical framework that includes social class when examining racism is important to consider in the development of future research and interventions.

Both the racialized working-class and racialized nonworking-class HCUs rated the same top 2 clusters high in importance for “race”/ethnic-based discrimination, indicating that within the health care setting, experiences of racism also occur *independent* of social class. Although there are limited empirical studies on social class,^23^ findings are consistent with Muntaner et al,^27^ who demonstrated that racism (and its impact on health) can occur independent of social class.

For the gender pattern match, racialized female HCUs had higher cluster ratings for “Racial/ethnic and class discrimination,” “Unequal access to health and health services,” and “Professional misconduct,” as compared to racialized male HCUs. On the cluster map, “Racial/ethnic and class discrimination” is located in the “Viewed as inferior” region, whereas the clusters “Unequal access to health and health services” and “Professional misconduct” are located in the “Unequal medical access” region. This finding suggests that for racialized female HCUs, “race”/ethnicity and gender are linked to their experiences of a lower quality of care and, in particular, unequal medical access.

This finding may also be a reflection of the current SEP/social class of racialized women in Toronto. Previous research had identified that in Canada, there is a racialization of poverty^16,17^ and a feminization of poverty; the 2006 Canadian national census data found racialized women are more likely to be unemployed and earn almost less than half the income of nonracialized men.^19^ In Toronto and the GTA, food bank visits have increased, with an overrepresentation of women and racialized groups.^68^ Taken together, these findings may be an acknowledgment of not only the difficulties in accessing medical care, but also the importance of addressing health needs and, accordingly, of racialized women maintaining the ability to work/stay employed in order to meet the financial challenges of paying for basic necessities such as food, shelter, and medication(s).

For the immigration status pattern match, as compared to racialized Canadian-born HCUs, racialized immigrant HCUs had a *lower* rating for the clusters “Negligent Communication” and “Unequal access to health and health services”; both clusters are found in the “Unequal medical care” region of the cluster map. This suggests that for racialized immigrant HCUs, access to health care is viewed as less of a concern, as compared to racialized Canadian-born HCUs. Although rates of poverty are higher for racialized recent immigrants,^16^ a possible explanation for the lower cluster rating may be that the majority of immigrants in this study were recent economic immigrants.

In Canada, economic immigrants are relatively healthier on arrival due to the mandatory medical examination required prior to entering the country.^69^ Indeed, a recent study found that economic immigrants did not use health services more than long-term residents or refugees.^70^ A systematic review identified that the “healthy immigrant effect,” whereby economic immigrants are in better health on arrival as compared to their Canadian-born counterparts, is stronger for more recent immigrants.^71^ If economic immigrants are in fact healthier on arrival, as suggested by this systematic review, this may explain why racialized immigrant HCUs did not rate clusters located in the “Unequal medical care” region of the cluster map as highly as Canadian-born HCUs. Specifically, accessing health care may not be as crucial toward maintaining health and, by extension, employment, because economic immigrant HCUs may be relatively healthier on arrival to Canada. Another reason may be that newer immigrants may not yet be aware of the exclusionary policies in Canada, as suggested by recent evidence from the Canadian Community Health Survey, the largest nationally representative data set, which found that the prevalence for perceived “race”/ethnic-based discrimination was significantly higher among *long-term* immigrants, as compared to newer immigrants.^72^

### Implications

Based on the findings from this study, there are several implications for HCPs and health care organizations. Findings indicate that racialized HCUs prioritized access to health. Yet, in experiencing racism in the health care setting, for some, this experience may result in delaying in or not seeking health care.^73^ To support access and quality of care, antiracist policies are needed. Translated into practice, an antiracist strategy focuses on both improving care by recognizing “race”/ethnicity as a social construct and addressing unequal power social relations. One way to minimize power imbalances is to tailor health care by assessing the patient's social determinants of health and providing medical care for racialized individuals and groups who self-define priorities.^74,75^

From the go-zone data, racialized HCUs identified that unequal medical care is also experienced through broader health care system policies. One priority, as identified by racialized HCUs for action/change based on “race”/ethnic-based discrimination, is “when a patient cannot get access to government-funded assist programs because of where the patient lives.” Current research suggests that this may be due to increasing racialized residential segregation based on income inequality within the GTA^76^ and the inconsistent provision of health services due to health care reform.^64^ To improve health care, policies are needed to provide more health care services in low-income areas and restore social programs/services in communities. Health care organizations and HCPs must advocate for services that can be accessed when and where people need. These priorities fall in line with those of the Ontarian population.^77^

Another priority identified is “when there is little or no access to language interpreters.” This finding indicates that currently, access to interpreters when receiving health care is not meeting the needs of racialized HCUs. The Institute of Medicine^38^ recognizes that language barriers in health care limit the understanding of a patient's medical condition and treatment, resulting in a lower quality of care, and consequently is a source of racial/ethnic inequities in health care. In terms of health care system obligations when it comes to language, the Ontario Human Rights Code prohibits discrimination on the grounds of “race” and ethnic origin, which is linked to language.^78^

HCPs must incorporate a broader systems thinking that includes acknowledging systemic racism in the labor market and the resulting, devastating impact of poverty on social conditions for racialized groups. In particular, this way of thinking brings into view the existence of an unequal, integrated system of policies and laws that result in racial/ethnic stratification or structural racism,^11^ which impacts the health and health care of racialized HCUs. Acknowledging this unequalness in system supports means recognizing that for racialized HCUs, health care services are not universally applicable or accessible. In this view, HCPs also have a moral obligation to address this injustice by advocating for improved health and social services for racialized communities.

There are several limitations to this study. CM activities were conducted in English; therefore, there is an absence of experiences specific to non-English-speaking racialized HCUs. Future studies should include interpreters to gain insight into additional mechanisms that contribute to classism and everyday racism in the health care system. Also, we conducted this study within a limited time frame, resulting in a smaller sample size of HCPs. Future studies with a larger sample size could examine the differences between racialized and nonracialized HCPs; and between different types of HCPs.

In terms of strengths in this study, the stress value for the cluster map is 0.18, indicating a good statistical fit. Another strength is that members of the Toronto and GTA communities participated in several phases of the research process, supporting a CM participatory approach. Additionally, as recommended, priority agenda setting for health care was determined by racialized HCUs—the community members negatively impacted by health care policies and practices.^53^

## Conclusions and Recommendations

This study contributes to the literature by identifying how classism and everyday racism occur in the health care system for some racialized HCUs; these discriminations occur through mechanisms of unequal power social relations in a context of institutional health care policies and practices such as cultural competence and a biomedical model of service delivery. Racialized HCUs reported “race”/ethnic-based discrimination and SEP/social class-based discrimination when receiving health care; these experiences differed based on social class, gender, and immigration status. Given that classism and everyday racism are interconnected for racialized HCUs in Toronto and the GTA, we conclude that to improve access to services and quality of care, antiracist policies—that focus on “race”/ethnicity as a social construct, unequal power social relations (classism and everyday racism), and a broader systems thinking—are needed to address institutional racism within the health care system.
